# Circular economy indicators: What do they measure?

**DOI:** 10.1016/j.resconrec.2019.03.045

**Published:** 2019-07

**Authors:** Gustavo Moraga, Sophie Huysveld, Fabrice Mathieux, Gian Andrea Blengini, Luc Alaerts, Karel Van Acker, Steven de Meester, Jo Dewulf

**Affiliations:** a Department of Green Chemistry and Technology, Ghent University, Coupure Links 653, 9000, Gent, Belgium; b Department of Green Chemistry and Technology, Ghent University, Graaf Karel de Goedelaan 5, 8500, Kortrijk, Belgium; c European Commission – Joint Research Centre, Sustainable Resources Directorate, Via E. Fermi 2749, 21027, Ispra, Italy; d Department of Materials Engineering, KU Leuven, Kasteelpark Arenberg 44, 3001, Leuven, Belgium

**Keywords:** Circular economy, Indicators, Sustainability, Life cycle thinking

## Abstract

Circular Economy (CE) is a growing topic, especially in the European Union, that promotes the responsible and cyclical use of resources possibly contributing to sustainable development. CE is an umbrella concept incorporating different meanings. Despite the unclear concept, CE is turned into defined action plans supported by specific indicators. To understand what indicators used in CE measure specifically, we propose a classification framework to categorise indicators according to reasoning on what (CE strategies) and how (measurement scope). Despite different types, CE strategies can be grouped according to their attempt to preserve functions, products, components, materials, or embodied energy; additionally, indicators can measure the linear economy as a reference scenario. The measurement scope shows how indicators account for technological cycles with or without a Life Cycle Thinking (LCT) approach; or their effects on environmental, social, or economic dimensions.

To illustrate the classification framework, we selected quantitative micro scale indicators from literature and macro scale indicators from the European Union ‘CE monitoring framework’. The framework illustration shows that most of the indicators focus on the preservation of materials, with strategies such as recycling. However, micro scale indicators can also focus on other CE strategies considering LCT approach, while the European indicators mostly account for materials often without taking LCT into account. Furthermore, none of the available indicators can assess the preservation of functions instead of products, with strategies such as sharing platforms, schemes for product redundancy, or multifunctionality. Finally, the framework illustration suggests that a set of indicators should be used to assess CE instead of a single indicator.

## Highlights

• Indicators are useful to support circular economy progress.
• Circular economy has different definitions entailing challenges for indicators.
• A classification framework to understand what indicators measure is proposed.
• Most of the analysed indicators focus on the preservation of materials.
• None of the analysed indicators focuses on the preservation of functions.

## 1 Introduction

Circular Economy (CE) is an approach to promote the responsible and cyclical use of resources. In recent years, CE has been endorsed as a policy to minimise burdens to the environment and stimulate the economy. CE is an umbrella concept (Blomsma and Brennan, 2017; CIRAIG, 2015; Homrich et al., 2018) that includes lowering material input and minimising waste generation (EASAC, 2016; EEA, 2016) to decouple economic growth from natural resource use (Cullen, 2017; EASAC, 2016; Pauliuk, 2018). Diverse nations are adopting CE around the world. The monitoring of CE at a macro scale currently includes methods using Material Flow Analysis (MFA), emergy analysis, and Input-Output analysis (Kalmykova et al., 2018). China was the first to enact a specific law in 2008 (CIRAIG, 2015); a large part of CE related literature refers to this country (Ghisellini et al., 2016; Homrich et al., 2018). Also, Germany and Japan were pioneers to promote CE in concrete policies (Geng et al., 2013). Late 2015, the European Union (EU) approved an action plan to implement CE across the union and member states (EC, 2015a). However, aspects of CE were already present in other EU policy, *e.g.* resource efficiency (EC, 2011) and waste related legislation developed since the 70 s (CEC, 1975). Recently, the European Commission (EC) proposed a monitoring framework on CE (EC, 2018a). Among private consultants, Ellen MacArthur Foundation (EMF) is in the roots of CE concept formulation (Ghisellini et al., 2016). Despite these actions, so far there is no commonly agreed concept of CE. Different actors have distinct interpretations of what CE could or should depict (Blomsma and Brennan, 2017), where the connection with sustainability is not always clear (Kirchherr et al., 2017).

Despite the blurred boundaries of CE definition, there is a need for specific methods to measure the CE progress. In this context, indicators can be useful in various implementation scales and as a tool to assess CE (EASAC, 2016; Geng et al., 2012). However, what to be measured in the sense of CE is subject for debate as the definition is ambiguous, and indicators might lead to different or even incoherent conclusions. Some authors reviewed tools and methodologies already in use. Elia et al. (2017) assessed a set of selected methodologies and indicators according to five CE characteristics^1^ deducted from the European Environmental Agency (EEA, 2016). The authors showed that none of the indicators and methodologies alone was capable of monitoring all the characteristics. Iacovidou et al. (2017) reviewed the methods to assess resource recovery from waste to promote CE. Their results showed that none of the methods alone could account for the retention of value in waste resources, and a holistic evaluation was necessary to encompass the environmental, economic, social, and technical dimensions of CE. Pauliuk (2018) proposed a dashboard of indicators to be used with the standard BS 8001:2017 from the British Standard Institute (BSI). This standard aims to help the CE implementation in businesses, organisations, and production systems; however, this standard does not contain compliance requirements (Pauliuk, 2018). The proposed dashboard used existing indicators to assess five characteristics promoted by the BSI standard (restore, regenerate, maintain utility, maintain financial value, and maintain nonfinancial value) and existing indicators for complementary characteristics (resource efficiency, climate, energy, and sufficiency).

Notwithstanding, the mentioned studies assessed CE tools considering restrictive CE characteristics (*i.e.* EEA and BSI) or restrictive scopes (*i.e.* resources recovered from waste). To bear CE as an umbrella concept, the classification of indicators has to consider CE encompassing different understandings. To our knowledge, such classification is yet to be made. The classification of existing CE indicators according to their capability can map the state of play for the development of new CE indicators. Hence, the objective of this paper is to understand *what quantitative indicators used to assess CE measure specifically, and how they do so.* This paper is limited to analyse output and outcome indicators according to the terminology given by Potting et al. (2017); thereon, we do not focus on input and throughput indicators. The aims are: (1) to propose a framework to classify indicators according to CE strategies (what) and measurement scopes (how) (Section 2); (2) to apply and discuss the framework with existing micro scale indicators from literature (Section 3); (3) to apply and discuss the framework with macro scale indicators using the European ‘CE monitoring framework’ as an example (Section 4). Finally, we present a closing discussion and conclusions (Section 5).

## 2 Establishing the classification framework

To establish the classification framework, we propose a rationale to clarify concepts in the CE context. In subsection 2.1 and 2.2, we present a rationale about critical topics for CE indicators. In Subsection 2.3, we present the framework overview.

### 2.1 Finding *what* indicators measure in CE

#### 2.1.1 CE definitions: *sensu stricto* and *sensu latu*

As an umbrella concept, CE is difficult to grasp. While some authors have proposed a consensual and broader definition (Kirchherr et al., 2017; Prieto-Sandoval et al., 2018), others have argued the attempt of a single definition is merely unachievable (Korhonen et al., 2018). We understand that by including only one CE definition, we potentially exclude possible meanings. However, to classify the indicators, we need to establish the boundaries encompassing the different CE approaches. As guidance, we use two definitions representing CE in *sensu stricto* and *sensu latu*.

The *sensu stricto* definition has a narrow focus. It is adapted from the rationale of Bocken et al. (2016) where CE is distinguished from the linear economy by two characteristics: slowing and closing resource loops. Slowing happens ‘through the design of long-life goods and product-life extension (*i.e.* service loops to extend a product's life, for instance through repair, remanufacturing),’ therefore ‘the utilisation period of products is extended and/or intensified, resulting in a slowdown of the flow of resources.’ Closing happens when ‘the loop between post-use and production is closed, resulting in a circular flow of resources,’ meaning the linear flows of waste are turned into secondary resources.^2^ Thereon, the *sensu stricto* focuses on the technological cycle of resources.

On the other hand, the *sensu latu* definition has a broader focus. It is given by Murray et al. (2017) where CE ‘is an economic model wherein planning, resourcing, procurement, production and reprocessing are designed and managed, as both process and output, to maximise ecosystem functioning and human well-being.’ Thereon, the *sensu latu* definition pushes the focus to sustainability and the effects CE strategies have on the economy, environment, and society. We do not discuss which definition (*sensu stricto* or *latu*) is more or less appropriate for CE, but we use the definitions as abasis to establish the framework to understand and map CE indicators.

#### 2.1.2 What to measure: CE strategies grouped by common aspects

CE strategies are largely defined in scientific and grey literature (Blomsma and Brennan, 2017). However, there is no consensual definition of each strategy promoting CE (Reike et al., 2017). For example, *reduce* can refer either to waste generation, raw materials input, eco-design (*e.g.* lightweight of products), or consumption. In this context, several ladders, or *R-frameworks*, position three or more strategies (Kirchherr et al., 2017). One *R-framework* uses ten strategies to increase circularity: refuse, rethink, reduce, reuse, repair, refurbish, remanufacture, repurpose, recycle, and recover (Potting et al., 2017). Despite the definitions, CE strategies can preserve products, their parts (modules and components), or the materials (and substances) present in each product’s part (Ghisellini et al., 2016; Iacovidou et al., 2017; Potting et al., 2017). Additionally, CE strategies can preserve the energy embodied in resources that cannot be preserved by other strategies (Kirchherr et al., 2017; Potting et al., 2017); landfilling and incineration for energy recovery should be used in the lack of other CE strategies.

CE strategies may also promote innovative business models that go beyond product preservation. Strategies for redundancy, multifunctionality, or use intensification of products promote CE by preventing the consumption of new products or creating new consumption patterns. For example, consumers may refuse to buy new products if services or multifunctional products create redundancy in the expected function (Potting et al., 2017). Renting, sharing, and pooling through Product Service-Systems (PSS) can be important instruments to promote CE because goods will be used in a more intensive way (Tukker, 2015). PSS can be oriented towards the product, use, and result (Kjaer et al., 2018; Tukker, 2015). Product-oriented PSS are related to additional services after the product sale (*e.g.* maintenance); thus, they focus on products. However, use- and result-oriented PSS focus on the preservation of the function provided by a product (Kjaer et al., 2018). For example, EMF (2015a) mentions sharing (such as car-sharing) and virtualisation (such as telemeetings instead of physical meetings) as CE actions; the first example is use-oriented, and the second example is result-oriented. In the case of product-oriented PSS, the strategies preserve the product, but in use- and result-oriented PSS, the strategies preserve the function.

All in all, the specific strategies in ladders can vary depending on the CE definition. Our aim is, rather than define specific strategies, to acknowledge the strategies’ capacity to promote CE considering common aspects. Hence, we propose a classification to group the existing CE strategies recommended by diverse authors. Inspired by the hierarchical ladder from Potting et al. (2018), we identified six common groups. The first-five groups acknowledge strategies for preservation, and the last group considers the reference scenario measurement. For the sake of simplicity, we call these six groups as CE strategies.

**Table:** 

Strategy 1	Preserve the **function** of products or services provided by circular business models such as sharing platforms, PPS (use- and result-oriented), and schemes promoting product redundancy and multifunctionality.
Strategy 2	Preserve the **product** itself through lifetime increase with strategies such as durability, reuse, restore, refurbish, and remanufacture.
Strategy 3	Preserve the product’s **components** through the reuse, recovery and repurposing of parts.
Strategy 4	Preserve the **materials** through recycling and downcycling.
Strategy 5	Preserve the **embodied energy** through energy recovery at incineration facilities and landfills.
Strategy 6	Measure the linear economy as the **reference scenario** or the absence of a preservation strategy to show the status, progress, or regress towards CE. For example, the indicator for waste generation per person in a year (EC, 2018a) might show whether the promotion of CE is generating less waste.

#### 2.1.3 Measurement type according to CE definition and CE strategies

CE does not work under a closed system. Circularity has direct and indirect effects on the economy (Potting et al., 2018). Its assessment can rely on direct and indirect indicators when data is unavailable, *e.g.* the proportion of PSS related to CE cannot be assessed yet, but indirect data from companies' report and surveys could provide a preliminary analysis (EEA - European Environment Agency, 2017). However, it is difficult to define what direct or indirect mean, since CE definition itself is debatable. To further address the problem, we propose that indicators may be direct or indirect in relation to the definition in sensu stricto or latu. In this way, both measurement types can be held in the classification framework without excluding views of CE in *sensu stricto* or *latu*. Moreover, Direct CE indicators may assess specific or non-specific strategies considering the rationale from subsection 2.1.2. Summarising, CE indicators can be classified into three measurement types:

a) Direct CE with Specific Strategies: indicators can focus on one or more identifiable CE strategies, *e.g.* Recycling Rate (Graedel et al., 2011) is specific to materials.
b) Direct CE with Non-specific Strategies: indicators always focus on more than one strategy, and it is not possible to recognise the explicit strategies, *e.g.* water withdrawal (Geng et al., 2012).
c) Indirect CE: indicators may evaluate aspects of CE strategies but with the use of ancillary approaches to assess CE, *e.g.* the indicator ‘Eco-innovation index’ form the Resource Efficiency Scoreboard (EC, 2016) rank European countries in relation to eco-innovation factors; the indicator may provide information on CE, but it is not direct to a CE definition.

### 2.2 Finding *how* indicators measure CE

#### 2.2.1 Measurement scopes according to Life Cycle Thinking (LCT) and modelling levels

**Fig. 1:**
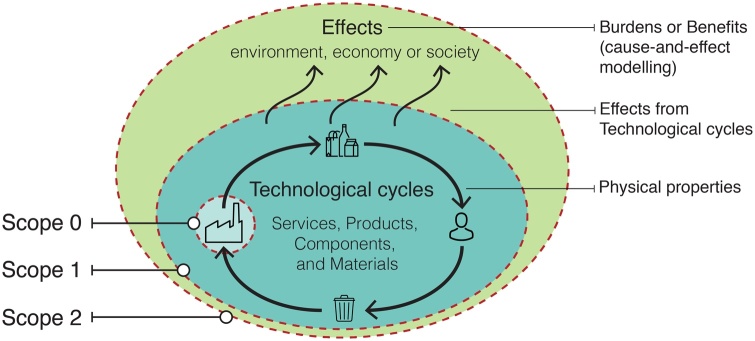
Proposed classification for the three measurement scopes from CE indicators.

CE acts on several steps of the production and consumption chain so that indicators may use a Life Cycle Thinking (LCT) approach. LCT is the capacity to look at products or services over the cycles of design, production, consumption, use, and disposal including interactions with sustainability (UNEP, 2005). LCT is considered as the state-of-the-art for analysing potential impacts (EC, 2003), and several reviews on CE show the necessity of a systemic view of the life cycle of resources (Ghisellini et al., 2016; Iacovidou et al., 2017; Reike et al., 2017). Moreover, LCT is at the heart of the Circular Economy Action Plan in the European Union that is actually split into sections concerning production, consumption, waste management, and production of secondary raw materials (EC, 2015a). Sustainability are divided in environmental, economic, social, and technical (technological) areas of concern (Dewulf et al., 2015). For the sake of simplicity in the cause-and-effect modelling, we consider that the technological cycles of materials, products, and services cause the effects on environmental, economic, and social domains (Fig. 1).

We argue that indicators measuring CE can be classified into three measurement scopes considering their LCT approach and modelling level (technological cycles and their cause-and-effect chain):

a) Scope 0: the indicators measure physical properties from the technological cycles without LCT approach, *e.g.* Recycling Rate (Graedel et al., 2011).
b) Scope 1: the indicators measure physical properties from the technological cycles with full or partial LCT approach, *e.g.* the indicator Reusability/Recyclability/Recoverability (RRR) in terms of mass includes the potential rate to reuse (products, components), recycle (materials), and recover (energy) (Ardente and Mathieux, 2014).
c) Scope 2: the indicators measure the effects (burdens/benefits) from technological cycles regarding environmental, economic, and/or social concerns in a cause-and-effect chain modelling, *e.g.* RRR benefit rate (RRR in terms of environmental effects) (Huysman et al., 2015).

#### 2.2.2 Implementation scale

CE has different implementation scales. The taxonomy from two reviews outlines three main scales: *micro* as a single product, company, or consumer; *meso* as eco-industrial parks and industrial symbiosis; and *macro* as a city, province, region, or nation (Ghisellini et al., 2016; Kirchherr et al., 2017). However, we have noticed that the *micro*, *meso*, and *macro* definition is neither consistently used nor clearly defined among different authors. The micro scale usually focuses on a single product, service, or organisation. The meso scale usually incorporates eco-industrial parks (Ghisellini et al., 2016). China is promoting industrial parks intensively (Geng et al., 2012), but they have specific characteristics: they integrate industrial, residential, business, research, and service areas (Geng and Doberstein, 2008). In this sense, the Chinese industrial parks are closely related to cities, indicating a macro scale. Additionally, Geng et al. (2012) also refer to meso as the development of networks beneficial to regions and the natural environment. The macro scale is usually limited to include the national level; where global can be an additional scale (EASAC, 2016). However, some authors suggest macro goes beyond countries, including the globe (CIRAIG, 2015; Kirchherr et al., 2017). Regions, with the scope between cities and countries, are considered macro scale for the Chinese CE law, but Smol et al. (2017) propose regions are the connection between macro and micro scales when measuring CE eco-innovation; indicating a meso scale. For the sake of understanding, we argue that the micro, meso, macro terminology should be followed by the specific range of the analysis (*e.g.* consumer, product, service, business, technology, city, park, region, nation, continent, or globe).

#### 2.2.3 Equation types of indicators

Generally, indicators are variables providing relevant information for decision-making (Gallopín, 1996). Variables are the representation of quantitative and qualitative attributes (Waas et al., 2014). Indicators can be either individual variables or a function of variables, *e.g.* ratio (number relative to a reference value), index (single number resulting from the aggregation of two or more variables), or the result of a complex simulation model (Gallopín, 1996). To *indicate*, indicators refer to a comparison value or reference (Waas et al., 2014). The reference value can be a baseline with undefined targets or baseline with specific (quantitative) or non-specific (qualitative) targets (Moldan et al., 2012). The reference can be either built-in or external to the indicator. To illustrate, the Sustainable Development Goal 12.3 aims to reduce by half the waste food per capita by 2030 (UN, 2015); an indicator for waste food could use the target as an external reference value (*e.g.* as parameters in a temporal evaluation) or as a built-in reference (*e.g.* as a ratio with the reference value as denominator).

The terminology used in this paper is following Sala et al. (2013) who made a clear distinction amongst methodology, method, model, and indicator. The CE evaluation has methodologies (*e.g.* LCA), which are a set of methods (*e.g.* LCA impact categories). A method groups models, tools, and indicators relevant for showing information on circularity (technological cycles or its cause-and-effect modelling). A model is a mathematical description of calculating an indicator, which can be obtained through a tool. An indicator is a variable (parameter) or a function of variables to provide information about circularity (technological cycles) or the effects (cause-and-effect modelling). Additionally, an indicator may be the result of the composite information on quantitative and qualitative data.

### 2.3 Classification framework

**Fig. 2:**
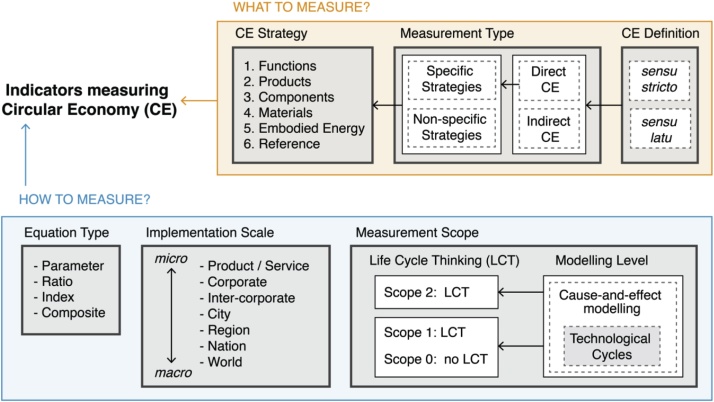
Classification framework for CE indicators.

The framework joins the rationale presented before for quantitative indicators (Fig. 2). CE strategies are grouped for the preservation of functions, products, components, materials, and embodied energy. Additionally, a reference scenario may be used for the assessment. The framework considers three scopes for the LCT approach: two measuring physical properties of the technological cycles (scopes 0 and 1), and one measuring the effects of the technological cycles (scope 2). The framework incorporates bio and non-bio materials; however, their cycles are treated equally. Once bio-based materials are inside economic cycles, they can be recovered by strategies with similar preservation focus as the non-bio materials, *e.g.* food composting is a downcycling process to recover nutrients, hence with focus on the materials; particleboards can be incinerated to recover the energy, hence with focus on the embodied energy.

## 3 Illustrating the classification framework: micro scale indicators

To illustrate the framework, we performed a literature review focusing on micro scale CE indicators. The review included three steps. (1) We searched on Scopus and Web of Science (WoS) databases the string indicator OR score OR metric OR measur* AND ‘circular economy’ in the title, abstract or keywords. We restricted the results by the English language and peer-reviewed documents. The Scopus database returned 251 results; the WoS returned 222 results. From the total, 154 were duplicates; hence 319 papers were analysed. (2) From a screening on title and abstract, we selected 11 documents proposing or discussing indicators for micro scale: products, services, and companies. (3) To the mentioned documents, we added three documents (grey and scientific literature) discussed by other authors. In total, we analysed 20 indicators from 14 documents. For all the selected indicators and equations, see the supplementary material.

In Section 3.1, we present a short overview of the framework illustrated with a set of indicators. In Section 3.2, we provide a critical analysis of the indicators and its classification.

### 3.1 Classification of the CE indicators: overview

**Fig. 3:**
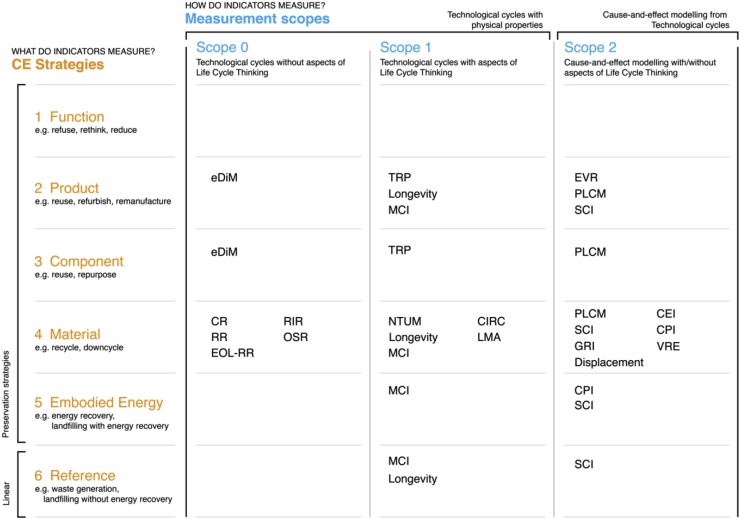
Indicators measuring CE at the micro scale. The found indicators encompass the measurement type of Direct CE with Specific Strategies. Indicators: eDiM (ease of Disassembly metric) from (Vanegas et al., 2018); CR (old scrap Collection Rate), RR (Recycling process efficiency Rate); EOL-RR (End of Life Recycling Rate); RIR (Recycling Input Rate); OSR (Old Scrap Ratio) from (Graedel et al., 2011); Longevity from (Franklin-Johnson et al., 2016); MCI (Material Circularity Indicator) from (EMF, 2015b); PLCM (Product-Level Circularity Metric) from (Linder et al., 2017); CPI (Circular economy Performance Indicator) from (Huysman et al., 2017); CEI (Circular Economy Index) from (Di Maio and Rem, 2015); VRE (Value-based Resource Efficiency) from (Di Maio et al., 2017); EVR (Eco-cost value ratio) from (Scheepens et al., 2016); NTUM (Number of Times of Use of a Material) from (Matsuno et al., 2007); CIRC (Material Circularity Indicator CIRC), TRP (Total Restored Products), LMA (Lifetime of Materials on Anthroposphere) from (Pauliuk, 2018); Displacement from (Zink et al., 2016); SCI (Sustainable Circular Index) from (Azevedo et al., 2017); GRI (Global Resource Indicator) from (Adibi et al., 2017).

Some patterns can be deducted from the framework illustration (Fig. 3). The measurement type of all analysed indicators is Direct CE with Specific Strategies because the indicators can discriminate the measured strategies. Considering the CE strategy, most of the indicators measure the preservation of materials - strategy 4. Considering the measurement scope, indicators are distributed mainly in scope 1 and scope 2 - they examine a partial or full LCT approach. Some indicators are in scope 1 by measuring more than one strategy of technological cycles, *e.g.* the Material Circularity Indicator (MCI) (EMF, 2015b) gauges properties in a product, components, materials, and potential waste generation. However, the measurement of more than one strategy is not a requirement for the classification in scope 1 or 2. The Lifetime of Materials in the Anthroposphere (LMA) (Pauliuk, 2018) and the Number of Times of Use of a Material (NTUM) (Matsuno et al., 2007) measure the cascading of materials over different product groups. The two indicators focus on recycling and downcycling to account for the residence time of materials; therefore, only strategy 4 is measured but LCT approach is achieved.

Finally, none of the analysed indicators measures strategy 1, which focuses on the preservation of functions. This CE strategy is achieved through dematerialisation of products with PPS, sharing platforms, products refusing through multifunctionality.

### 3.2 Classification of the CE indicators: analysis

#### 3.2.1 Indicators focusing on functions

Although none of the reviewed indicators assesses functions, some of them attempt to measure functions using a composition of quantitative and qualitative indicators. For example, Scheepens et al. (2016) used the Eco-costs Value Ratio (EVR) (a quantitative LCA-based indicator) and the Circular Transition Framework (a qualitative structure) to assess a PSS for water tourism. While the EVR provided an analysis of the products used in the PPS, the qualitative framework focused on the required steps for the PSS implementation. It is unclear what was the function-related strategy; the assessment analysed the substitution of a PPS with a diesel engine by a PPS with an electrical engine. In this sense, the EVR provided the eco-design improvement in the product used by the service, but the preservation of functions is not clearly depicted.

The preservation of functions is not as straightforward as the other strategies. The comparison between services and products demands the attention on specific aspects of CE, such as the consequences caused by consumers' behaviour change (Zink and Geyer, 2017). The Circularity Gap report (Wit et al., 2019) provides insights into what could be the evaluation of functions in the global scale. The authors used Material Flow Analysis (MFA) and a Sankey diagram to show the transformation from natural and secondary resources into ‘societal needs’ (*i.e.* housing, communication, mobility, healthcare, services, consumables, and nutrition) in one year. Methodologies such as LCA and MFA may evaluate the preservation of functions, but indicators are still necessary.

#### 3.2.2 Indicators focusing on products and components

The indicators measuring strategies on products or components consider at least the *sensu stricto* aspect to slow resources loops. Indicators measure this aspect in several ways, but two ways deserve attention: the assessment of quantity and quality. Indicators measuring quantity can account for tangible properties that are not user- or market-related. For example, the Total Restored Products (TRP) (Pauliuk, 2018) is MFA-based; it accounts for the products reused, refilled, refurbished, redistributed, and remanufactured at the end-of-life (EoL). On the other hand, indicators measuring quality account for properties influenced by the user or markets, such as time or economic value. For example, the Product-Level Circularity Metric (PLCM) (Linder et al., 2017) is a ratio from the economic value from recirculated flows over the economic value of all flows. Another one measuring quality is the Longevity indicator (Franklin-Johnson et al., 2016); it uses lifespan estimations from statistical records and experts approximation to account for the duration of materials in products. In contrast with the Longevity indicator, the results from PLCM can be equal for similar products with different lifespans (products with identical function and recirculated flows). However, the Longevity indicator by only including the average lifespan has to deal with the data variability caused by different consumer behaviour. Additionally, the Material Circularity Indicator (MCI) (EMF, 2015b) uses information on mass (virgin and recycled materials and waste) and product lifespan in one index system. For the moment, quantity information may be more reliable, but quality is a measure that deserves the attention of indicators in CE and may show the influence of consumer behaviour.

#### 3.2.3 Indicators focusing on materials, embodied energy and the reference scenario

From our analysis, it is clear that most of the indicators focus on strategies to preserve materials. This result was expected because CE has a high emphasis on recycling (Ghisellini et al., 2016). Recycling is the most frequent strategy across different CE concepts (Kirchherr et al., 2017). The indicators measuring materials consider at least the *sensu stricto* aspect to close resource loops. However, it is not possible to identify a pattern showing how materials’ preservation is measured. The indicators can gauge information based on different characteristics of materials, *e.g.* supply and demand interactions (Displacement indicator from Zink et al., 2016), or the creation of economic value (Circular Economy Index from Di Maio and Rem, 2015).

Additionally, the illustration seems to point out that authors developing CE indicators at the micro scale are less concerned with the preservation of embodied energy and the assessment of waste generation. Energy recovery is usually understood as the last preferred option (EMF, 2013; Potting et al., 2017). However, the Circular economy Performance Indicator (CPI) shows that for plastics the options should depend on the material quality; if the waste quality is low, recycling may result in higher environmental impacts than incineration (Huysman et al., 2017). In any case, both recycling and energy recovery are neither green nor burden-free (Allwood, 2014). Furthermore, CPI and MCI can account for energy recovery; and MCI can account for unrecoverable waste.

#### 3.2.4 Composite indicators

Finally, some indicators use a composition of qualitative and quantitative information to assess CE. For example, the Sustainable Circular Index (SCI) for manufacturing companies from Azevedo et al. (2017) considers sustainability reports (Triple Bottom Line, Global Reporting Initiative, and others) and the MCI. SCI includes the weighting of the information with factors determined by a panel of experts. Another composed indicator is the Global Resource Indicator (GRI) from Adibi et al. (2017). GRI combines scarcity, geopolitical availability, and recyclability. The scarcity and recyclability are quantitative measures of resource availability, recycling rate, and dissipative losses. Geopolitical availability is a qualitative parameter for the geopolitical stability of the countries where the resource is available, and a parameter for the homogeneity of distribution.

## 4 Illustrating the classification framework: ‘CE monitoring framework’ in the European Union

To illustrate the framework with macro scale indicators, we selected the indicators recently proposed by the European Commission (EC, 2018a). The EC proposal is one possible example of CE indicators at macro scale; other examples could include the proposals from the Netherlands (Potting et al., 2018), France (Magnier et al., 2017), or China (Geng et al., 2012) with recent efforts to provide emergy-based indicators (Geng et al., 2013).

In Section 4.1, we present an overview of the framework illustration. In Section 4.2, we provide a critical analysis of the indicators classification.

### 4.1 Classification of the ‘CE monitoring framework’: overview

The CE monitoring framework is the EC proposal for measuring CE progress in the EU and Member States (EC, 2018a). The ‘CE monitoring framework’ divides indicators into four topics: production and consumption, waste management, secondary raw materials, and competitiveness and innovation. Those are closely related with the priority areas from the CE Action Plan in Europe: plastics, food waste, critical raw materials, construction and demolition, and biomass and bio-based products (EC, 2015a). The EC proposal presents ten indicators, but six of them also have so-called ‘sub-indicators.’ In total, the proposal uses twenty-four measurement guides. The indicators are based on existing information from Eurostat, the Raw Materials scoreboard, and the Resource Efficiency scoreboard (EC, 2018a).

**Fig. 4:**
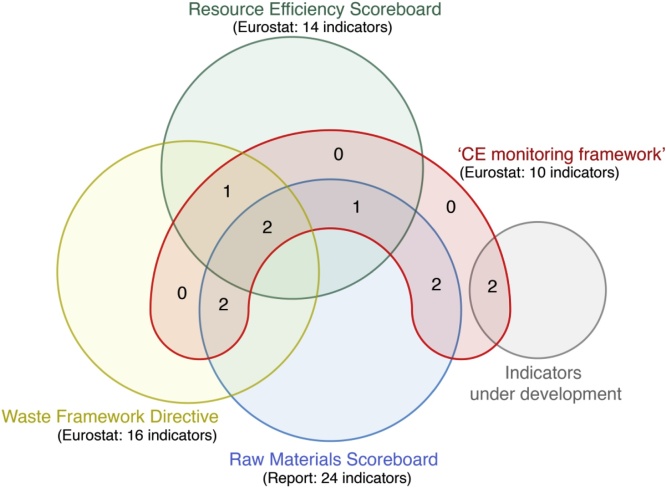
The interaction of the indicators from the ‘CE monitoring framework’ and other European directives shows that the indicators are not unique to the ‘CE monitoring framework’.

Eight indicators from the ‘CE monitoring framework’ are present in other European frameworks^3^ and are not unique to CE (Fig. 4). The other indicators are under development: ‘Food Waste’ and ‘Green Public Procurement’ (GPP). In any case, the measurement of the first was foreseen in the revision of the EU Waste Directive (EC, 2015b). For the GPP, data are still unavailable. GPP significance for CE may depend on the inclusion of relevant requirements (*e.g.* reparability, durability, and recyclability) in public contracts and procurements (EC, 2018a). Both indicators are also in the scope of the Sustainable Development Goals for responsible consumption and production (EC, 2018a).

The ‘CE monitoring framework’ also uses material flow analysis (MFA) with Sankey diagrams to give an overview of materials flows in the EU. The diagrams show aggregated information of metallic and non-metallic materials, fossil energy, and biomass providing an initial guide for a more detailed MFA. The diagrams may be used to extract indicators for CE, but at this point, the ‘CE monitoring framework’ does not describe those specific indicators. For this reason, the Sankey diagrams are not analysed in this paper.

According to the classification framework, the eight available indicators from the ‘CE monitoring framework’ mainly focus on materials - strategy 4 (Table 1). Four indicators are Direct CE with Specific Strategies; the other four are Indirect CE indicators. The Indirect CE indicators may concern to specific strategies, but they measure CE with ancillary aspects; not fitting in the *sensu strictu* or *latu* definition. Within the four Direct CE indicators, nine sub-indicators are measuring only materials, one is measuring both materials and components, and three are measuring the reference scenario. All those Direct CE indicators measure mass properties.

**Table 1:** Classification of the indicators proposed by the European Commission to measure the CE development. Strategies inside brackets mean the indicator contains aspects of that measurement. All Direct CE indicators are ‘Direct CE with Specific Strategies.’

Indicator	Sub-indicator	Strategy	Scope	Measurement type
1. Self-sufficiency for raw materials	–	[4]	0	Indirect CE
2. Green public procurement	–	Indicator not available		
3. Waste generation	Generation of municipal waste per capita	6	0	Direct CE
	Generation of waste per GDP	6	0	Direct CE
	Generation of waste per DMC	6	0	Direct CE
4. Food waste	–	Indicator not available		
5. Recycling rates	Recycling rate of municipal waste	4, [6]	0	Direct CE
	Recycling rate of all waste	4, [6]	0	Direct CE
6. Recycling / recovery for specific waste streams	Recycling rate of overall packaging	4, [6]	0	Direct CE
	Recycling rate of packaging waste by type	4, [6]	0	Direct CE
	Recycling rate of wooden packaging	4, [6]	0	Direct CE
	Recycling rate of e-waste	3, 4, [6]	1	Direct CE
	Recycling of biowaste	4, [6]	0	Direct CE
	Recovery rate of C&D waste	4, [6]	0	Direct CE
7. Contribution of recycled materials to raw materials demand	End-of-life recycling input rates	4	1	Direct CE
	Circular material use rate	4	1	Direct CE
8. Trade in recyclable raw materials	Imports from non-EU countries	[4]	2	Indirect CE
	Exports to non-EU countries	[4]	2	Indirect CE
	Imports from EU countries	[4]	2	Indirect CE
	Exports to EU countries	[4]	2	Indirect CE
9. Private investments, jobs and gross value added	Gross investment in tangible goods	[2, 3, 4, 6]	2	Indirect CE
	Number of persons employed	[2, 3, 4, 6]	2	Indirect CE
	Value added at factor cost	[2, 3, 4, 6]	2	Indirect CE
10. Patents related to recycling and secondary raw materials	Patents of recycling and secondary materials	[4]	2	Indirect CE

The ‘Recycling rate’ and ‘Recycling and recovery for specific waste streams’ do not consider LCT approach (scope 0) except by one ‘sub-indicator’ in scope 1 (Recycling rate of e-waste), because of the inclusion of market and End of Life (EoL) information. Furthermore, the ‘Contribution of recycled materials to raw materials demand’ clusters two sub-indicators in the scope 1: ‘End-of-life recycling input rates’ (EoL-RIR) and ‘Circular material use rate.’ Finally, the indicator ‘Waste generation’ monitors the amount of waste as a reference scenario to close material loops.

### 4.2 Classification of the ‘CE monitoring framework’: analysis

#### 4.2.1 Direct CE with specific strategies indicators

Our classification framework shows that the Direct CE indicators from the ‘CE monitoring framework’ focus mainly on measuring material and waste production. Material resources and waste are considered the primary focus of the European policy on CE (McDowall et al., 2017). The specific indicators show that the EU has an understanding of CE similar to the *sensu stricto* definition but mostly restricted to the circulation of materials. The indicators ‘Recycling rate,’ ‘Recycling for specific waste streams,’ and ‘Contribution of recycled materials to raw materials demand’ monitor the loops of materials by measuring the quantity of recycling and secondary materials. Those indicators gauge closed-loop and open-loop systems without differentiation, meaning that recycling and downcycling are accounted in the same way. Some authors argue the distinction between loops is unnecessary; closed-loops do not always displace more primary material than open-loops, and closed-loops may promote dispersive applications (Geyer et al., 2016). In any case, the measurement of the quality, or how much the loop of materials displace the primary production is relevant to CE (EEA, 2016; Moriguchi, 2007). The ‘Contribution of recycled materials to raw materials demand’ addresses the contribution of recycling to raw materials demand. Its sub-indicators do not consider quality, but the approach can indicate the displacement of materials in general mass terms.

Waste generation is an inevitable outcome of any economic activity due to entropy creation (Georgescu-Roegen, 1973), but changes in waste generation may indicate changes in consumption patterns (EC, 2018a). However, those changes may also be a result of other structural variations rather than CE promotion (EEA, 2016). The indicator ‘waste generation’ introduces the idea of waste decoupling. In this context, decoupling refers to decrease in waste generation per gross domestic product (GDP) or per domestic material consumption (DMC) unit. Particularly, the idea of resource and environmental decoupling is not present in the ‘CE monitoring framework’. Resource decoupling is an intermediate objective from the European CE (Ghisellini et al., 2016), and it is included in the Resource Efficiency scoreboard as the lead indicator.

Besides the evaluation of materials and waste, the ‘CE monitoring framework’ has one specific sub-indicator accounting for the reuse of components in waste of electrical and electronic equipment (WEEE). Differently from the other recycling and reuse rate sub-indicators, that only measure the EoL phase, this sub-indicator measures also the quantity of electrical and electronic equipment (EEE) entering the market (EC, 2018a). Despite WEEE information being critical to recovering resources, WEEE policy usually promotes weight-based targets considering neither resource types, quality, nor production steps (*e.g.* metallurgy) (UNEP, 2013). Furthermore, the complexity in EEE products determines the possibility of recycling, but the current EEE design tends to complexity (Graedel and Reck, 2014), which difficult their preservation. In a general manner, the ‘CE monitoring framework’ does not capture the assessment of products or information on products design, but the indicator on WEEE is a step forward.

The EU recognises the design of products as a fundamental CE aspect (EC, 2015a, 2015b). However, the ‘CE monitoring framework’ puts the indicators for self-sufficiency, green procurements, waste generation, and food waste under the categorisation of ‘production and consumption;’ those do not assess products or services in a specific way. According to the European Economic and Social Committee (EESC, 2018), the ‘CE monitoring framework’ misses relevant indicators in eco-design and CE business models. Products and services are central to slowing resources loops (Bocken et al., 2016), but the ‘CE monitoring framework’ does not capture the role of the consumer in the flow of resources (EESC, 2018). The EU has a strong focus on eco-design policy (*e.g.* Eco-Design Directive), but this experience is not yet present in their CE indicators. It is worth mentioning that macro scale data on products do not exist for the EU context; the information on durability, lifetime, disassembly, repair, and reuse cannot be monitored at this moment (EEA, 2016). However, not always product-related strategies are a priority in the EU policy, *e.g.* the EU policy for plastics acknowledges the reuse of products as low importance because it ‘is only an option for a limited number of waste streams’ (EC, 2018b). Moreover, how to correlate micro and macro scale indicators is not yet presented in this current version of the EU monitoring framework; however, this is a shortcoming of the current literature. Arnsperger and Bourg (2016) reflected this lack of relation in micro/macro scales could lead companies to become more circular but not the economy. All in all, the inclusion of CE requirements in green public procurements, is promising for increasing data availability. Despite not covering the whole economy, public procurements represent over 14% of the European GDP (EC, 2017); they might be the most accessible path to assess products and services.

#### 4.2.2 Indirect CE indicators

Indirect CE indicators from the ‘CE monitoring framework’ mainly focus on materials or aspects from materials, strategy 4. The indirect indicators measure ancillary aspects of CE, showing awareness of relevant areas, but not necessarily encompassing circularity. For example, the indicator for the number of patents related to recycling does not consider the quantity or quality of secondary materials being produced nor its effects. It uses registered patents as a ‘proxy for technological progress’ (EC, 2018a). Innovation and technology support CE progress but are not objectives of the *sensu stricto* or *latu* definitions. Equally, the indicator for the trade of secondary raw materials shows the fluxes of materials considering a country’s border but not necessarily CE requirements. Despite evaluating materials, the cause-and-effect chain of how trade affects the increasing of recycling may exist, but it is not documented by the ‘CE monitoring framework.’ Differently from the recycling rates, that show the EU commitment to increase the recycling potential from waste, trade supports the dynamicity of the EU market (EC, 2018a). Primary motivations for international trade rely on recycling costs and advantages (*e.g.* countries with less restrictive environmental laws for recycling) (Beukering et al., 2014). Trade explains the international demand and supply of secondary materials, but its impact on recycling is ambiguous and challenging to summarise across different materials (Beukering, 2001). Additionally, assuming a positive correlation between international trade and CE, it is also necessary to understand how illegal trade, not tracked by the indicator, influences the result.

Another indicator, the ‘self-sufficiency for raw materials’ is linked with the security of supply of raw materials in critical sectors indicating a key role for recycling actions, in particular when self-sufficiency is very low (EC, 2018a). However, considering the indicator also accounts for primary production, a country may increase self-sufficiency with mining; then self-sufficiency is also a measure for the linear economy. Indeed, Europe is self-sufficient in construction minerals and wood because of domestic extraction (JRC-EC, 2016). Recycling is directly correlated with self-sufficiency (*i.e.* increasing materials recycling means increasing self-sufficiency); however, it is not an indicator measuring circularity of materials (EESC, 2018). Moreover, decreasing self-sufficiency can indicate the increased risk of supply disruptions, or that other-policy measures are achieving decoupling in the EU at the expenses of other countries from which more raw materials are imported.

Additionally, the ‘private investments, jobs, and gross value added’ related to CE sectors shows the effects of CE considering products, components, materials and waste. The indicator and its sub-indicators account for investments, employment, and share of GDP in 24 NACE codes (Statistical Classification of Economic Activities in the European Community) identified by the EU as proxies for recycling, repair, and reuse (EC, 2018a). The selected NACE codes cover diverse sectors, *i.e.* waste collection and trade, scrap trade, second-hand retail, components retail, dismantling, and maintenance and repair of industrial and household equipment (EC, 2018a). However, the NACE classification was not created to bear or distinguish CE activities (Ketels and Protsiv, 2017). For example, lifespan increase can be virtually applied to any product, but it is not possible to include all industries with actions to increase lifespan using the NACE codes. Finally, ICE indicators do not encompass CE main objectives, but their track can help governmental responses to promote CE if results are critically analysed.

## 5 Closing discussion and conclusion

In this paper, we presented a classification framework for CE indicators and used it to evaluate *what quantitative indicators used to assess CE measure specifically, and how they do so*. This section aims to present the strengths and weakness of the classification framework (5.1) and the conclusion of the framework illustration with the contribution for policymaking (5.2). For the specific discussion of the framework illustration, check the subsections 3.2 and 4.2.

### 5.1 Strengths and weakness of the classification framework

The framework classifies indicators by common CE strategies (what) and measurement scopes (how) according to Life Cycle Thinking (LCT) approach. The presented approach is a novel way to categorise indicators without being restrictive to specific definitions. Hence, the framework highlights the inherent characteristic of CE as an umbrella concept. We argue CE has different strategies that can be distinguished in five preservation groups (function, product, component, material, and embodied energy) and one group to measure the linear economy as a reference scenario. CE as buzzword creates confusion that entails challenges for the selection and development of appropriate CE indicators. Our proposal has the added value to differentiate CE indicators by the measurement approach independent of the definition of CE, either in *sensu stricto* or *latu*. At this point, the framework cannot differentiate indicators measuring inputs and outputs; *e.g.* indicators for the total amount of recycled material (output) and the total investment in recycling activities (input) are part of the same strategy group. Both input and output are necessary to evaluate CE transition, but the framework still needs refining to include input indicators consistently.

Additionally, the classification framework includes three scopes considering the LCT approach. The scopes present an initial proposal to differentiate the possible mechanisms behind the cause-and-effect chain in CE. CE includes at least the circularity of materials, components, and products, but CE may also include effects on the economy, environment, and society. The relation amongst all these concerns is complex. The relation type between CE and sustainable development varies as conditional, beneficial, and having trade-offs that may also lead to adverse outcomes (Geissdoerfer et al., 2017). The cause-and-effect chain of how CE affects sustainable development is not fully documented, where in other areas this is clearer, *e.g.* climate change has a well-documented impact pathway from the pollutant emission to the impact on areas of protection. For the sake of simplicity, we address the problem detailing two scopes for technological cycles (one without LCT approach and one with LCT approach) and one for the effects of technological cycles over the other sustainability concerns. The added value of the mentioned approach is the ability to easily differentiate *how* indicators measure the progress to CE. Future work may include an extended definition of how LCT approach is treated in scope 2 - effect of the technological cycles. Moreover, Fig. 2 summarised the rationale behind CE indicators. However, at the present point, CE indicators are too heterogeneous, and we do not have evidence of a pattern to identify interrelationship amongst the presented aspects (*e.g.* Equation Type *versus* Implementation Scale *versus* Measurement Scope); this could be better explained in future studies.

Additionally, some authors argued CE assessment includes the use of renewable energy, water, and land (EEA, 2016; Elia et al., 2017; Geng et al., 2012). For example, Elia et al. (2017) called as CE requirement the ‘increase share of renewable and recyclable resources,’ including renewable energy; Ellen MacArthur Foundation quoted ‘replacing fossil fuels with renewable energy’ as an example for the principles behind CE (EMF, 2015a). The (lack of) consideration of non-material flows is one of the critiques from CE; not all authors engage in the same interpretation (Blomsma and Brennan, 2017). Notwithstanding, our classification framework is designed to include the measurement of non-material flows. Energy and water, for example, influence all CE strategies, and their indicators fit the framework under the *sensu latu* definition as specific or non-specific strategies.

### 5.2 Conclusion and contribution for policymaking

To illustrate the classification framework use, we applied it with micro scale indicators (products, businesses, and companies) and macro scale indicators (from the European ‘CE monitoring framework’). From the analysed studies, it is possible to conclude that most indicators focus on the preservation of materials. Strategies focusing on materials, especially recycling, are well-developed actions but they are some of the existing options to promote CE: recycling even being essential to the economy is not the only aspect of a sustainable CE. According to the European Economic and Social Committee (EESC, 2018), all indicators from ‘CE monitoring framework’ are ‘heavily focused on waste’as result of the reliability on waste data and lack of other options. Our classification framework can complement the EESC opinion. On the one hand, the indirect CE indicators contain aspects of waste and materials information. On the other hand, the direct CE indicators based on recycling rates use waste data to provide information on the possible preservation of materials^4^ . The recycling rates from the ‘CE monitoring framework’ are a promise that a fraction of waste will be *upgraded* as a secondary resource. In this regard, what may be important in the materials side of the ‘CE monitoring framework’ is that only a fraction of the waste prepared for recycling will turn into a recycled material whereas efficiency and quality of those materials and processes are not yet covered.

Additionally, none of the analysed indicators seems to focus on functions, such as multifunctionality or product sharing. Notwithstanding, we argue existing methodologies, such as LCA and MFA, can provide a starting point for the assessment of functions. Those methodologies still need to deal with practical issues to evaluate CE. For example, diverse authors recommend LCA to evaluate CE (Elia et al., 2017; Lonca et al., 2018; Scheepens et al., 2016); but some CE strategies (such as recycling, reuse, repurposing, multifunctionality, or co-production) are in the scope of unsolved problems in the LCA methodology (Bobba et al., 2018; Reap et al., 2008). Furthermore, the evaluation of functions is challenging because it induces changes in consumer behaviour, *e.g.* sharing platforms may motivate a less-careful use of products when compared to ownership (Tukker, 2015). High-level CE strategies demand socio-institutional changes in the product chain, increasing the complexity of the evaluation (Potting et al., 2017). Moreover, the definition of the specific strategies for the preservation of functions still needs clarification, *e.g.* which type of PSS promote CE. The classification framework shows the preservation of functions as an open question for CE indicators. Although the less clear boundary of functions preservation (compared to products or materials) may also increase uncertainty in CE evaluation.

Lastly, the application of the framework seems to suggest that not one, but a set of indicators are necessary to assess CE. None of the analysed CE indicators measures all preservation strategies directly, *i.e.* CE includes many dimensions, and one indicator would hardly be able to summarise them all. In a similar sense, a set of indicators is promoted by CE monitoring systems on macro scale, *e.g.* Europe and China, and micro scale, *e.g.* Pauliuk (2018) and EMF, 2015b when the optional complementary indicators are considered for the second. Moreover, CE might promote sustainable development; hopefully, future discussion and in particular the ISO technical committee for CE (ISO/TC 323) will shed light on the subject.

## Disclaimer

The authors declare no competing financial interests. This publication contains the opinions of the authors, not that of the Flemish administration or the European Commission. The Flemish administration and the European Commission will not carry any liability with respect to the use that can be made of the produced data or conclusions.

## Footnotes

1 According to Elia et al. (2017): Reducing input and use of natural resources; reducing emission levels; reducing valuable materials losses; increase share of renewable and recyclable resources; and increasing the value durability of products.

2 In this paper, closing (resource loops) is used as a reference to prevent waste generation, as far as possible, in the post-use phase. It does not preserve the product or components and includes open/closed loop recycling, downcycling, and energy recovery.

3 Waste-related indicators from Eurostat are considered proxies for the Waste Framework Directive.

4 Recycling rate is the ratio of the waste prepared for recycling activities by the total amount of the waste stream.
